# Anatomical description of the vertebrobasilar and carotid systems forming the cerebral arterial circle in the equine brain

**DOI:** 10.3389/fvets.2026.1772131

**Published:** 2026-03-24

**Authors:** Ahmad Al Aiyan, Abdulrahman Fahad Alnahdi, Sara Abu Hayah, Arwa Alshamsi, Hessa Alshebli, Sara Aleissaee, Rinsha Balan

**Affiliations:** Department of Veterinary Medicine, College of Agriculture and Veterinary Medicine, United Arab Emirates University, Al Ain, United Arab Emirates

**Keywords:** carotid arterial system, cerebral arterial circle, Circle of Willis, equine brain, vertebrobasilar system

## Abstract

**Introduction:**

The cerebral blood supply in horses is maintained by carotid and vertebrobasilar systems, which join the cerebral arterial circle to ensure continuous brain perfusion. This study aimed to comprehensively describe the origins, courses, and interconnections of the vertebrobasilar and carotid systems in the horse brain.

**Methods:**

Ten adult equine specimens were obtained from horses euthanized for non-neurological reasons. Following perfusion with 10% formaldehyde, colored latex was injected into the arterial system, and detailed dissections were performed to study major intracranial vessels.

**Results:**

The vertebral and ventral spinal arteries merge to form the basilar artery, which courses rostrally along the ventral brainstem, giving rise to medullary, pontine, and cerebellar branches before joining the caudal communicating arteries. The carotid system enters the cranial cavity through the internal carotid arteries, which give rise to the rostral and middle cerebral arteries rostrally and the caudal communicating arteries caudally. Both systems form a complete cerebral arterial circle, ensuring extensive collateral circulation. Minor variations were noted in the origin and caliber of cerebellar arteries, and in the presence of a rostral communicating artery; however, the circle remained functionally intact in all specimens.

**Conclusion:**

These findings confirm that unlike ruminants, horses lack a carotid rete mirabile and retain patent internal carotids and a functional basilar artery, providing dual cerebral inflow that ensures vascular redundancy and stability, an adaptation for equine neurovascular physiology and comparative anatomy.

## 1 Introduction

Given the high metabolic demand and limited energy reserves of neurons, a continuous and well-regulated supply of arterial blood is essential for the central nervous system. In mammals, this blood supply is secured through complex arterial networks that ensure collateral circulation, thereby reducing the risk of ischemia or stroke during vascular obstruction (1, 2). The cerebral arterial circle, also known as the Circle of Willis, serves as the primary anastomotic hub, integrating blood from multiple afferent systems before distribution to the brain tissue (3). The pattern of cerebral blood supply varies across species. In most ruminants, the extracranial internal carotid arteries regress postnatally, and the brain is supplied mainly through the rostral epidural rete mirabile, with minimal contribution from the basilar artery, which often carries blood away from the circle (2, 4). In contrast, camelids maintain both the internal carotid artery and rete while preserving the vertebrobasilar input (3, 5, 6). However, horses differ markedly from ruminants in that they lack the carotid rete mirabile, retaining fully developed internal carotid arteries, and their vertebral arteries fuse into a basilar artery that delivers blood rostrally into the circle of Willis (7, 8).

This places equine cerebral circulation closer to that of carnivores and primates, where the carotid and vertebrobasilar systems are essential contributors (1, 7). Within the equine brain, two arterial systems support cerebral perfusion: the carotid system, which supplies the forebrain, including frontal, parietal, and temporal lobes; and the vertebrobasilar system, which supplies the hindbrain, cerebellum, and brainstem (7, 9).

The carotid system originates from the common carotid arteries and includes the internal carotid arteries that enter the cranial cavity via the foramen lacerum and join the arterial circle. The rostral and middle cerebral arteries arise from these, supplying cortical and subcortical regions (8, 9). The vertebrobasilar system forms by merging vertebral and ventral spinal arteries to create the basilar artery, which ascends along the ventral brainstem before contributing to the circle and giving rise to the cerebellar arteries (1, 5, 6).

Despite the importance of these systems in equine neurophysiology and clinical relevance in conditions like cerebrovascular accidents and guttural pouch mycosis, detailed studies on horse brain arterial architecture remain limited (9). Reports highlight variability in internal carotid artery branching patterns, including unusual anastomoses with occipital or basilar arteries; however, systematic descriptions remain scarce (9, 10). Comparative data from ruminants, camelids, and carnivores highlight diverse cerebrovascular arrangements among mammals. However, the equine configuration remains unclear regarding afferent sources, arterial circle organization, and anatomical variations.

This study aimed to provide a comprehensive analysis of the two main cerebral arterial systems in horses, specifically the vertebrobasilar and carotid systems. By delineating the anatomical components, areas of perfusion, interconnections within the cerebral arterial circle, and observed variations, this study aimed to enhance our understanding of equine cerebrovascular anatomy. This knowledge is fundamental for veterinary neuroanatomy and clinical diagnostics and comparative investigations into the evolution of mammalian cerebral circulation.

## 2 Material and methods

Adult equine brains were obtained from 10 mixed-breed horses (four mares and six geldings) with no history of neurological disorders. The horses were euthanized for reasons unrelated to this study. The horses were aged 10–20 years. The fatal illnesses that led to euthanasia were diverse but did not involve the central nervous system. All specimens were handled and transported to the Department of Veterinary Medicine at the United Arab Emirates University under institutional biosecurity guidelines.

### 2.1 Fixation and vascular injection

Upon arrival at the laboratory, the common carotid arteries were carefully exposed, and cannulas were inserted into both common carotid arteries. The vascular system was gently flushed with a physiological saline solution to remove residual blood clots. Subsequently, 10% neutral buffered formaldehyde solution was injected via both common carotid arteries. The specimens were maintained at a controlled temperature of 5 °C for 24 h. The following day, a colored latex solution (neoprene latex mixed with red pigment) was manually injected into the arterial system using 50–60 ml syringes to fill the arterial system. The arterial injection procedure followed protocols previously described and validated by our group in detailed cerebrovascular studies (11–14). Latex was injected manually under slow, steady hand pressure rather than mechanical pumping, allowing tactile control and minimizing the risk of vascular rupture. Injection was continued until consistent resistance was encountered and distal arterial filling was visually confirmed, indicating adequate perfusion of the major intracranial vessels. After the injection, the heads were skinned and thoroughly cleaned of superficial tissues to provide better access to the cranial cavity. All specimens demonstrated symmetric and homogeneous latex filling of the principal cerebral arteries, and no brains were excluded due to incomplete perfusion. Each specimen was immersed in a container containing 10% neutral buffered formaldehyde solution for a minimum of 2 weeks to achieve proper fixation of both neural and vascular tissues.

### 2.2 Dissection, observation, and documentation

The skulls were accessed using a rotating oscillating saw, followed by a dorsal craniotomy. The dura mater was meticulously incised to allow careful extraction of the brain and its arterial supply, ensuring that it remained intact. Special attention was paid to preserving the arterial circle and major afferent vessels. The vertebrobasilar and carotid circulatory systems were traced meticulously from their origins to the intracranial branches. The courses of the vertebral, basilar, and internal carotid arteries and their branches, which contribute to the cerebral arterial circle, have been documented. The arterial architecture was examined macroscopically in all specimens. High-resolution digital photographs were obtained using a DSLR camera with macro settings. Image analysis was performed using Adobe Photoshop software (version 2025; Adobe Inc., San Jose, CA, United States).

## 3 Results

### 3.1 Gross anatomical observations

**Figure 1:**
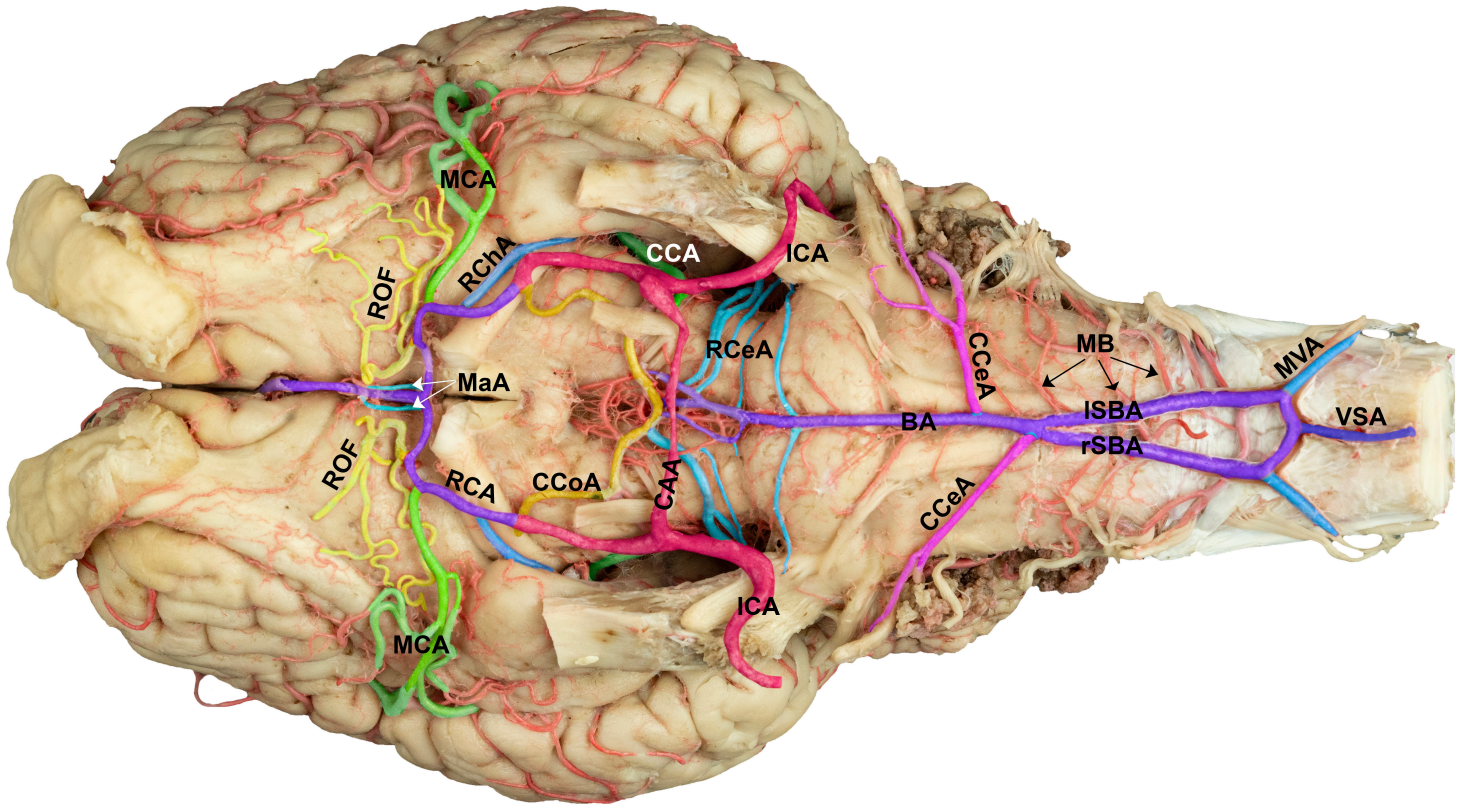
Ventral view of the arterial supply to the horse brain, illustrating the vertebrobasilar and carotid systems. The complete ventral surface of the horse brain is shown, with all major arteries supplying the brain highlighted to demonstrate the two principal cerebral arterial systems: the vertebrobasilar and carotid systems. The vertebrobasilar system originates caudally from the ventral spinal artery (VSA) and medial branches of the vertebral arteries (MVA), which unite to form the right (rSBA) and left (lSBA) segments that converge rostrally to form the basilar artery (BA). The BA gives rise to the medullary branches (MB), caudal cerebellar artery (CCeA), and rostral cerebellar artery (RCeA), which supply the brainstem and cerebellum. Rostrally, the carotid system contributes via the internal carotid artery (ICA), which forms the caudal communicating artery (CCoA), rostral cerebral artery (RCA), middle cerebral artery (MCA), rostral choroidal artery (RChA), marginal artery (MaA), and rostral olfactory artery (ROF). The caudal anastomotic artery (CAA) connects the two internal carotid arteries.

Examination of the ventral surface of the equine brain revealed two principal arterial systems: the vertebrobasilar and carotid systems. These systems join at the brain base to form the cerebral arterial circle, ensuring collateral circulation and continuous neural perfusion (Figure 1).

### 3.2 Vertebrobasilar system

**Figure 2:**
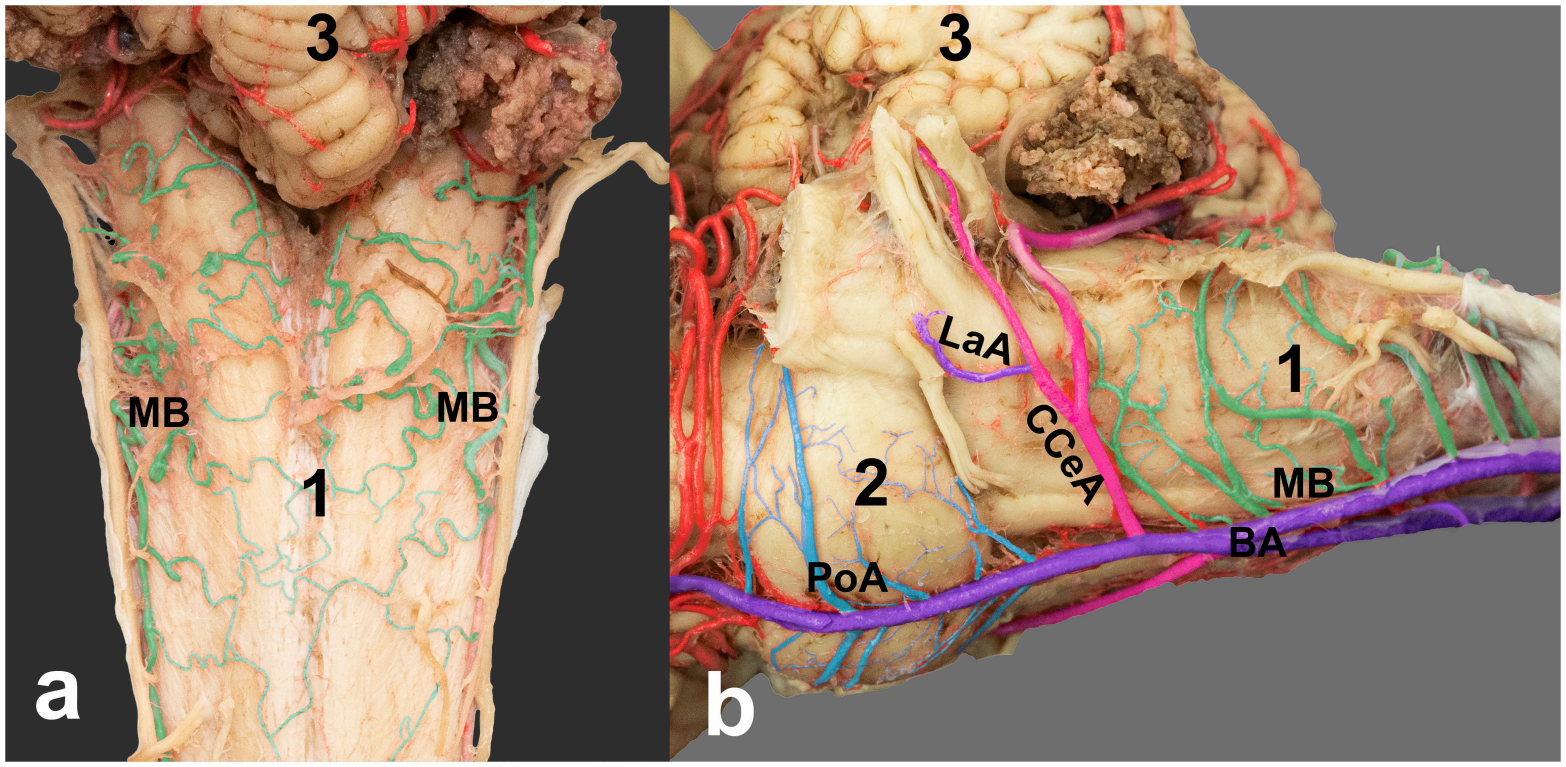
Vascular anatomy of the medulla oblongata and pons in the horse brain. **(a)** Dorsal view and **(b)** left ventrolateral view showing the basilar artery (BA) and its major branches in the caudal aspect of the brain. Medullary branches (MB, colored light green), pontine arteries (PoA, colored light blue), caudal cerebellar artery (CCeA), and its branch, the labyrinthine artery (LaA). 1, medulla oblongata; 2, pons; 3, cerebellum.

The vertebrobasilar system is formed caudally by the ventral spinal and paired vertebral arteries. The ventral spinal artery arises from the spinal cord region and courses dorsorostrally, joining the medial branches of the vertebral arteries at the medulla oblongata (Figure 1). The vertebral arteries, originating from the subclavian arteries, ascend through the transverse foramina of the cervical vertebrae and enter the cranial cavity through the magnum foramen, forming lateral and medial vertebral branches. At the level of the spinal roots of the nervus accessorius, the medial branch of the vertebral artery and ventral spinal arteries fused on each side, forming right and left segments of the basilar artery, which traveled together for varying distances while giving rise to prominent branches to the medulla oblongata before fusing to form the basilar artery (Figures 1, 2b). Although the fusion sites differed, the most frequent location was approximately halfway along the length of the medulla oblongata. The two basilar artery segments give rise to a series of medullary branches as they traverse the length of the medulla oblongata. These medullary branches traverse tortuously over the curved body of the medulla to supply the lateral and dorsal aspect of the medulla oblongata (Figures 2a, b).

After the two basilar segments unite to form the main basilar artery, the basilar artery gives rise to the right and left caudal cerebellar arteries. These arteries course dorsolaterally toward the caudal aspect of the cerebellum, supplying blood to the lateral and caudal portions of the cerebellar hemispheres and the vermis (Figures 1, 2b). The caudal cerebellar artery gave rise to the labyrinthine artery, which coursed laterally toward the internal auditory meatus in association with the vestibulocochlear nerve, supplying the inner ear and the adjacent choroid plexus (Figure 2b). Near the rostral border of the pons, the basilar artery gave rise to three to four roots creating paired rostral cerebellar arteries. These vessels ascended dorsolaterally to supply the rostral cerebellar hemispheres. The basilar artery continued rostrally until joining the caudal communicating arteries, contributing to the cerebral arterial circle (Circle of Willis; Figure 1). Variations occurred in the caliber and branching patterns of the rostral cerebellar arteries in several specimens. Occasional duplication of the initial roots, ranging from three to five, was observed. Minor asymmetries between the left and right caudal cerebellar arteries were present, with one side forming a more dominant trunk in three of the 10 brains examined. In five samples, one or two roots on one or both sides originated from the caudal communicating artery, besides those arising from the basilar artery (Figure 1).

### 3.3 Carotid system

**Figure 3:**
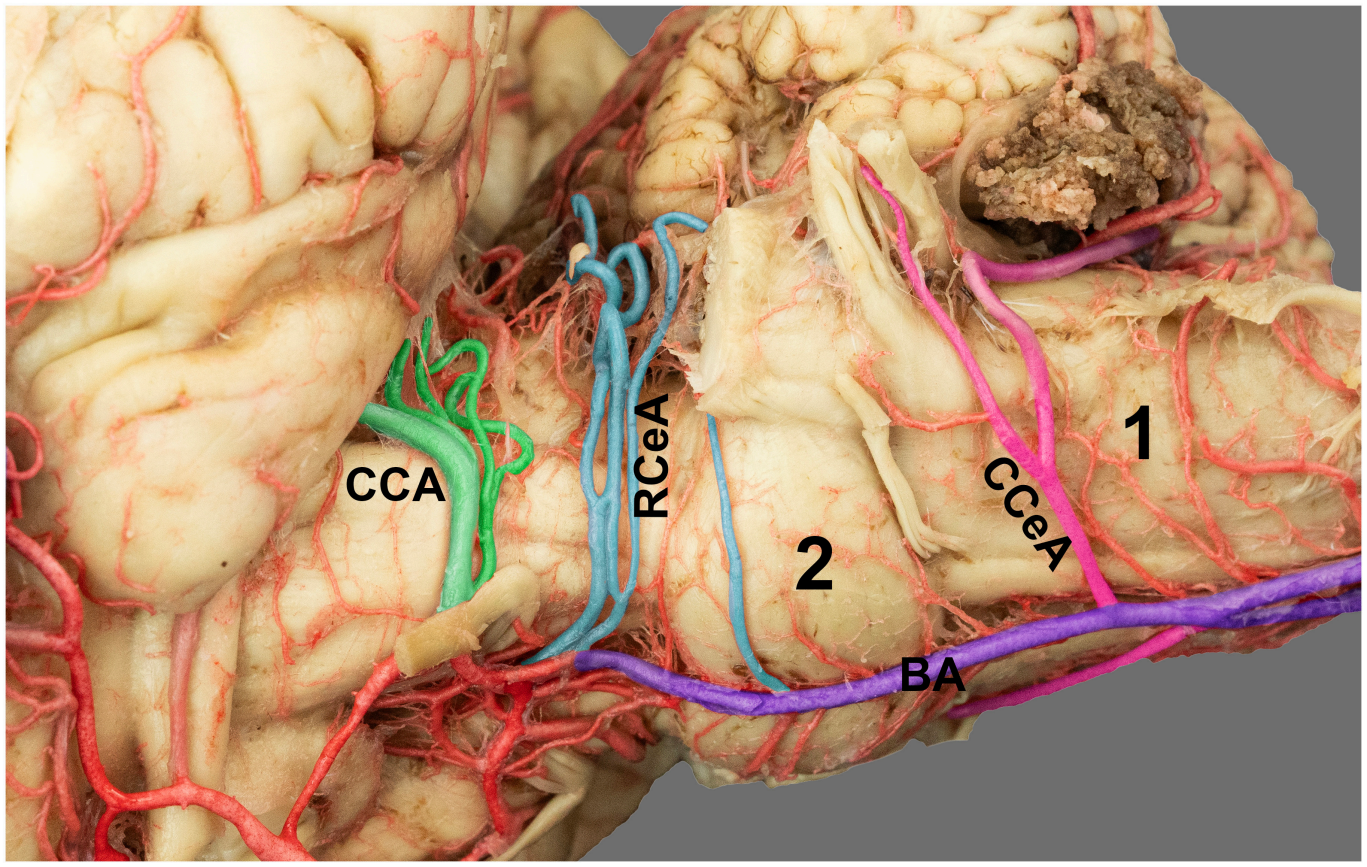
Lateroventral view of the horse brain showing the arterial supply to the brainstem and cerebellum. The basilar artery (BA) runs along the ventral midline of the brainstem; the caudal cerebellar artery (CCeA) curves dorsolaterally toward the caudal cerebellar hemisphere; the rostral cerebellar artery (RCeA) ascends toward the rostral cerebellar area; and the caudal cerebral artery (CCA) extends toward the caudal aspect of the cerebrum. 1, medulla oblongata; 2, pons.

The carotid system arose from paired common carotid arteries, which bifurcated into the external and internal carotid branches near the occipital artery. The internal carotid artery enters the cranial cavity via foramen lacerum, courses rostrally and dorsally, and contributes significantly to the lateral and rostral quadrants of the cerebral arterial circle. Along its intracranial course, the internal carotid artery gives rise to the caudal communicating and rostral cerebral arteries (Figure 1). The rostral cerebral artery travels rostrally and gives the rostral choroidal artery, which travels along the medial surface of the piriform lobe and hippocampal sulcus and then curves dorsally to supply the choroid plexus of the lateral ventricle. The middle cerebral artery arises from the rostral cerebral artery at the base of the lateral surface of each hemisphere, curves laterally around the pyriform lobe, and passes through the Sylvian fissure (Figure 1). Then, it divides into several branches that supply the lateral surfaces of the frontal, parietal, and temporal lobes. After leaving the middle cerebral artery, it joins its contralateral counterpart at the rostral midline, completing the rostrolateral portion of the arterial circle. At that level, the rostral cerebral arteries frequently give rise to rostral olfactory branches supplying the olfactory bulbs and tracts (Figure 1). The rostral cerebral artery travels rostromedially through the longitudinal fissure, issuing cortical branches to the medial surfaces of the frontal and parietal lobes. The caudal communicating artery extends caudally from the internal carotid artery and joins the basilar artery within the interpeduncular fossa (Figure 1). Along its course, it gives rise to the caudal cerebral artery, which extends dorsomedially over the corpus callosum and supplies the caudomedial occipital cortex while giving choroidal branches to the caudal horn of the lateral ventricles (Figures 1, 3). Minor variations were noted in the origin of the caudal cerebral artery. In four horses, the caudal cerebral artery arose as two roots from the caudal communicating artery. The caudal cerebral artery, in its dorsomedial course, gives off several small branches to supply the dorsal surface of the brainstem.

### 3.4 Cerebral arterial circle

**Figure 4:**
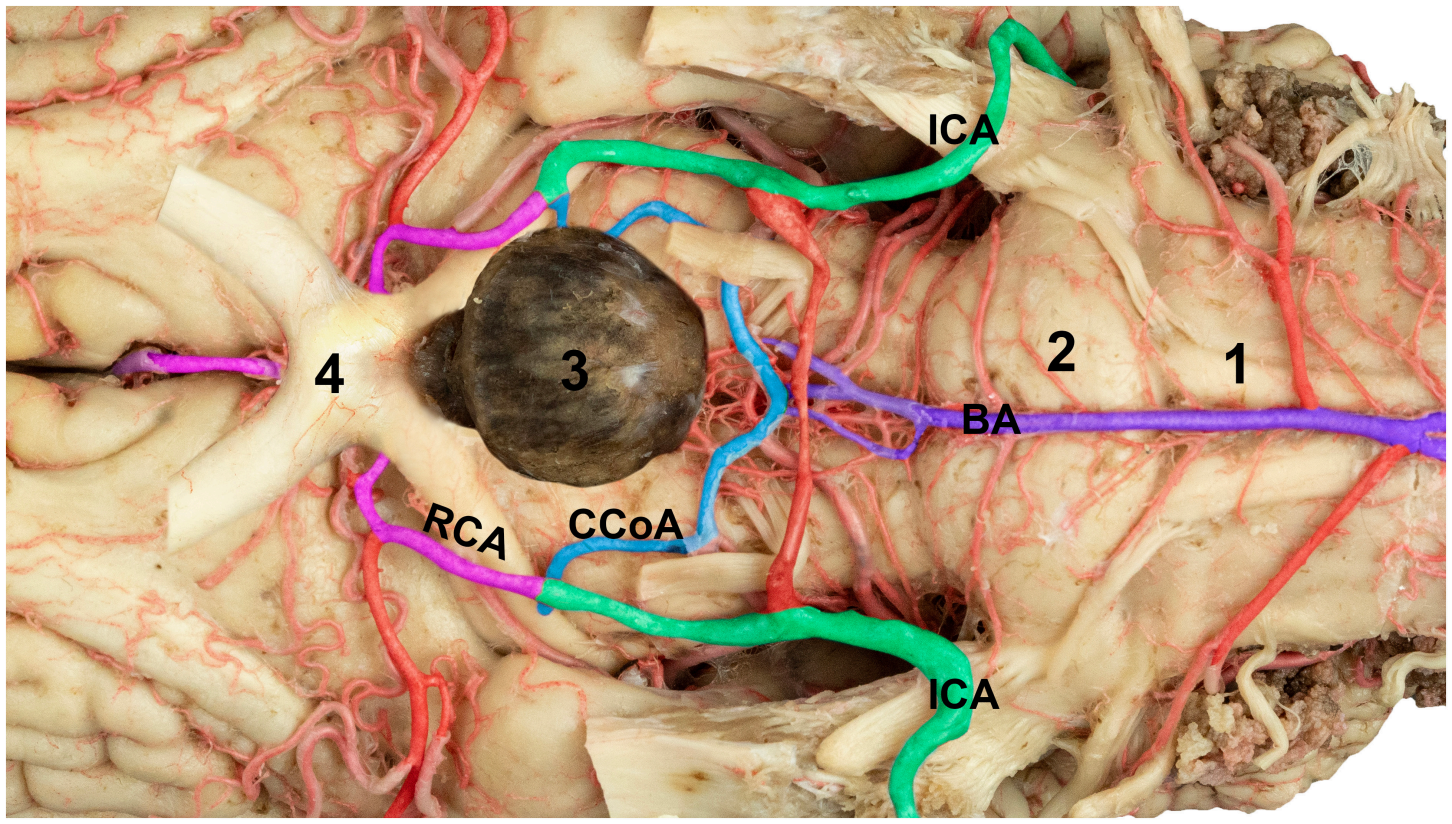
Magnified *ventral* view of the ventral surface of the horse brain showing the arteries forming the cerebral arterial circle. ICA, internal carotid artery; RCA, rostral cerebral artery; CCoA, caudal communicating artery; 1, medulla oblongata; 2, pons; 3, pituitary gland; 4, optic chiasm.

The cerebral arterial circle (Circle of Willis) forms a complete arterial polygon surrounding the optic chiasm, the mammillary body, and the pituitary gland on the ventral surface of the brain (Figures 1, 4). It is formed by paired rostral cerebral arteries rostrally, caudal communicating arteries laterally and basilar arteries caudally. This configuration enables blood convergence from the vertebrobasilar and carotid systems, ensuring a collateral supply to the major divisions of the brain.

The rostral communicating artery, connecting the paired rostral cerebral arteries, was present in seven of the 10 studied brains, forming a short transverse connection. In contrast, it was absent in three specimens, leaving the anterior part of the circle open. In addition, a slight asymmetry was observed in the size of the caudal communicating arteries, with the right side being larger than the left in four cases. Despite these variations, the arterial circle remained functionally continuous in all the specimens, indicating an anastomosis between the vertebrobasilar and carotid systems (Figures 1, 4). A summary of the observed anatomical variations and their occurrence across the 10 examined specimens is presented in Table 1.

**Table 1:** Summary of observed anatomical variations in the cerebral arterial system of 10 equine specimens.

**Anatomical variation**	**Description**	**No. of specimens (10)**
Rostral communicating artery	Present	7
Rostral communicating artery	Absent	3
Asymmetry of caudal communicating arteries	Right larger than left	4
Caudal cerebral artery origin	Two roots from caudal communicating artery	4
Caudal cerebellar artery asymmetry	One side forming dominant trunk	3
Rostral cerebellar artery origin	Additional roots from caudal communicating artery	5

## 4 Discussion

This study demonstrates that the equine brain receives blood supply from two major systems, the carotid and vertebrobasilar arteries, which merge in the cerebral arterial circle. The carotid system, represented by internal carotid arteries and their branches, perfuses the forebrain and most of the cerebrum. In contrast, the vertebrobasilar system, formed by vertebral and ventral spinal arteries uniting the basilar artery, supplies the brainstem and cerebellum before joining the arterial circle. This anatomical configuration provides the structural basis for redundant arterial inflow and potential collateral circulation between the carotid and vertebrobasilar systems (8, 15).

Physiological studies in equids have demonstrated substantial internal carotid artery contribution to cerebral perfusion, supporting the anatomical basis for dual arterial inflow described here (16).

Although the present study is limited to anatomical observations, the completeness of the cerebral arterial circle and dual afferent supply suggests a capacity for collateral flow, as supported by comparative and clinical literature.

Our findings align with those of previous studies in horses. Moraes et al. (15) reported that the basilar artery arises from the vertebral arteries, with caudal cerebellar arteries as its first branches, followed by pontine and rostral cerebellar arteries. We observed similar branching patterns and variability in the cerebellar arteries between specimens. Moraes et al. (15) described differences in the basilar artery size and branching between males and females. However, our sample size was insufficient to evaluate sex-based differences.

These findings align with those of Böing et al. (17), who demonstrated that the middle cerebral artery typically arises from the rostral cerebral artery, forming multiple cortical branches supplying the outer surface of the brain. We confirmed the dominant role of the MCA in supplying the lateral surfaces of the frontal, parietal, and temporal lobes, whereas the rostral cerebral artery supplied the medial cortical regions. Variations in MCA branching were noted, consistent with the findings of Böing et al. (17).

Angiographic studies in equids have documented variability in internal carotid artery and carotid–basilar anastomoses (18). Earlier anatomical studies in horses have also reported direct carotid–basilar connections, supporting the persistence of dual cerebral inflow pathways in this species (19).

In our series, irregular duplication of the rostral cerebellar artery and asymmetry of the caudal cerebellar arteries were observed, showing that the equine cerebrovascular system exhibits inter-individual variability. In cattle, sheep, and goats, the extracranial internal carotid artery regresses postnatally, and the intracranial segment is reconstituted via the rostral epidural rete mirabile fed by maxillary artery branches (4, 20, 21). Consequently, the carotid system dominates cerebral inflow in these species, whereas the basilar artery tapers caudally and carries blood away from the circle of Willis (2, 22). Our findings in horses contrast sharply with this arrangement: the internal carotids remain patent, and the basilar artery contributes directly to the cerebral supply.

The anatomical variations observed in this study may have clinical relevance in equine neurological disease and diagnostic imaging. Variability in cerebellar artery roots and asymmetry of the caudal communicating arteries could influence regional perfusion patterns and collateral flow. In addition, the presence or absence of a rostral communicating artery may affect interhemispheric redistribution of blood during unilateral arterial obstruction. Although functional consequences were not assessed, recognition of these variants is important for the interpretation of angiographic studies and post-mortem evaluation of cerebrovascular lesions in horses.

Camelids exhibited intermediate conditions. Like ruminants, they possess a rete mirabile and retain a functional internal carotid artery and a basilar artery that actively contribute to the circle (3, 5, 6, 23). This dual input resembles a horse more closely than a sheep. Similarly, the absence of a carotid rete in mouse deer indicates that a complete internal carotid artery persists in some primitive ruminants, resembling equine conditions (24). Cervids, including fallow deer and reindeer, conform to the ruminant model, relying on the rete mirabile for cerebral inflow with limited vertebrobasilar contributions (25, 26). Functional interpretations emphasize thermoregulation via the rete, which is absent in horses. In carnivores, primates, and horses, the carotid and vertebrobasilar systems supply the circle of Willis (1, 22). This pattern represents a more generalized mammalian condition in contrast to the specialized arrangement in ruminants.

The dual contributions of the carotid and vertebrobasilar systems in horses provide several functional advantages. First, a complete cerebral arterial circle ensures collateral circulation, thus enabling compensation during arterial occlusion (15). This may be relevant in equine conditions like guttural pouch mycosis, where internal carotid compromise occurs. Secondly, the absence of a carotid rete suggests that selective brain cooling, an adaptation in ruminants and camelids, is not a primary in the equine cerebrovascular system (27, 28). Horses may rely on other thermoregulation mechanisms. From an evolutionary perspective, the horse's retention of patent internal carotids and the afferent basilar artery represents a primitive mammalian condition, as highlighted by comparative studies across vertebrates (29).

The development of a rete in most artiodactyls represents a derived specialization for thermoregulation and pulse dampening (22, 30). Horses, like carnivores and primates, retain ancestral conditions, with a cerebrovascular pattern that ensures redundancy and high perfusion pressure to the brain. This study was limited by the number of specimens examined, which restricted the assessment of sex- and age-related differences and precluded population-level conclusions regarding the frequency of observed anatomical variations. In addition, the present study relied exclusively on gross anatomical dissection following latex injection. Although this approach is well-suited for descriptive vascular anatomy, complementary techniques such as angiography or micro-computed tomography could provide three-dimensional visualization and allow more precise assessment of vessel caliber and spatial relationships (6). Given the descriptive aim of this work and the use of latex-based gross dissection, quantitative measurements were not considered sufficiently reliable for formal analysis. Future studies incorporating standardized angiography, corrosion casting, or micro-computed tomography would be better suited for precise morphometric and three-dimensional vascular quantification.

## 5 Conclusion

This study demonstrates that the equine brain receives its blood supply from two major arterial systems: the carotid and vertebrobasilar arteries. The internal carotid arteries contribute through the rostral and middle cerebral arteries, whereas the vertebral and ventral spinal arteries unite to form the basilar artery, which forms cerebellar arteries and joins the cerebral arterial circle. The complete Circle of Willis ensures robust anastomosis between both systems, providing effective collateral circulation across brain regions. In contrast to ruminants, in which the carotid rete mirabile dominates, and the basilar artery carries minimal blood to the brain, horses retain patent internal carotid arteries and functional basilar arteries. This pattern more closely resembles that of camelids, donkeys, carnivores, and primates, underscoring the evolutionary diversity of mammalian cerebrovascular organizations. Dual supply in horses likely confers functional advantages in maintaining cerebral perfusion under conditions of vascular compromise. By providing a detailed anatomical description of the vertebrobasilar and carotid systems in horses, this study addresses a gap in equine neuroanatomy. These findings are relevant to veterinary neurobiology, clinical practice, and comparative anatomy, and offer a framework for understanding cerebrovascular variations and their implications for health and disease.

## Data Availability

The raw data supporting the conclusions of this article will be made available by the authors, without undue reservation.
